# Editorial: Microbial Source Tracking

**DOI:** 10.3389/fmicb.2021.795564

**Published:** 2021-11-25

**Authors:** Michèle Gourmelon, Anicet R. Blanch, Georg H. Reischer

**Affiliations:** ^1^IFREMER, ODE-DYNECO-Pelagos, Laboratoire d'Ecologie Pélagique, Plouzané, France; ^2^Department Genetics, Microbiology, and Statistics, University of Barcelona, Barcelona, Spain; ^3^Institute of Chemical, Environmental, and Bioscience Engineering, TU Wien, Vienna, Austria

**Keywords:** microbial source tracking, fecal pollution indicators, microbiota (16S), bacterial communities, molecular marker, water, bacteriophages, modeling

## Introduction

Human and animal fecal pollution may adversely affect inland and coastal waters with negative consequences for water supplies, recreational water uses and shellfish production. Fecal pollution of waters is a significant health risk and can lead to economic losses due to shellfish bed closures, bathing prohibitions and serious limitations on water resources. Fecal contamination in water is currently evaluated by the enumeration of traditional fecal indicator bacteria (FIB; i.e., *Escherichia coli* and intestinal enterococci) which are not indicating the fecal pollution source. Microbial source tracking (MST) methods allow the identification of fecal pollution sources that is critical for management and remediation of water quality.

This special issue in *Frontiers in Microbiology* section *Microbiotechnology* offers the collection of 19 original research manuscripts, which contribute to the current knowledge on the microbial source tracking and highlight the latest developments.

## MST Approaches

A large number of MST methods have been developed targeting bacteria, viruses, chemical compounds and eukaryotic cells. Many of them demonstrate an appropriate host specificity and sufficient sensitivity. Culture-dependent and culture-independent (mainly molecular) methods have been proposed. With the exception of chemical compounds, they are considered in the manuscripts of this special issue.

Multiple varying sources of fecal pollution can influence water quality. Some widespread sources of fecal pollution such as discharges of untreated or treated wastewater, contamination by bathers and wild birds are investigated in beach waters (Li et al.; Sinigalliano et al. and Toubiana et al.), street-level stormwater run-off (Monteiro et al.) and in roof-harvested rainwater (Denissen et al.) in this Research Topic.

MST methods used in the studies collected in this issue are based on the search for bacterial molecular markers, the detection of phages and the application of bacterial community approaches. More specifically, the most frequently used methods are bacterial molecular markers (in particular based on the 16S ribosomal RNA sequence or 16S rDNA gene). Those molecular markers targeting human fecal material, or pollution originating from livestock or birds are the methods of choice in at least 15 of the manuscripts in this current Research Topic. In an interesting trend, most studies looked for several sources and combined several methods and targets (e.g., phage detection, bacterial molecular marker quantification, bacterial communities analysis).

The 16S rDNA gene is also the target of bacterial community-based MST approaches by amplicon sequencing of this gene. Boukerb et al. used this approach to identify novel markers for avian and other sources. Liang et al. as well as Zimmer-Faust et al. applied the same method to investigate and model fecal pollution in rural rivers and coastal waters, respectively, combining it with PCR-based detection of host-associated MST markers.

Rytkönen et al. explored the utility of targeting 16S rRNA instead of 16S rDNA as a template in MST studies. Furthermore, manuscripts of this Research Topic have shown that it may be relevant to complement the results from MST methods with chemical parameters indicative of pollution sources. Sinigalliano et al. used isotopic tracking of nitrogen inputs while Reynolds et al. investigated correlations of ammonium, total oxidized nitrogen, and phosphorus levels with MST marker levels.

Highly relevant technical issues of MST investigations were explored by Byappanahalli et al. who investigated the influence of filter pore size on marker- and community-based MST approaches while Linke et al. demonstrated the strong impact inorganic turbidity can have on DNA based water quality analysis. These studies highlight the central importance of quality and process controls in MST investigations.

## MST and Health Risks—Tracing Pathogens and Antimicrobial Resistance

If the use of MST markers is currently coupled with the detection of FIB, it is also done in combination with the detection of human pathogens, especially in the context of quantitative microbial risk assessment (QMRA). Denissen et al. investigated the presence of *Mycobacterium tuberculosis, Yersinia* spp. and *Listeria monocytogenes* in roof-harvested rainwater. Derx et al. used *Cryptosporidium* and *Giardia* as index pathogens in a catchment wide QMRA approach.

MST methods are also used to trace and investigate the spread of antimicrobial resistant strains and the pollution they originate from in the environment. Ahmed et al. show that MST markers can be useful indicator for the presence of AMR genes while Toubiana et al. combined genetic MST markers with AMR gene detection to assess bathers as sources of contamination.

## Viral and Phage Targets

Ballesté et al. used host-associated bacteriophages as indicators for human pathogenic viruses. Kapoor et al. on the other hand used bacteriophages as a low-cost alternative for differentiate animal waste and human wastewater in Kolkata. Viral indicators together with genetic MST markers were also used as an input for a QMRA investigation by Kongprajug et al.

## MST Prediction and Modeling

Wu et al. used various machine learning approaches to predict sources of fecal pollution based on a broad set of parameters from land use to hydrological parameters. In another study, Green et al. used generalized linear mixed models and conditional inference trees to find predictive variable for pollution source in 68 streams in the Finger Lakes region of Upstate New York.

## Conclusion and Perspectives

The selection of MST markers for their combined use with each other or with other markers such as chemical ones, is becoming a useful methodological approach to determine sources of fecal contamination and improve the robustness of prediction models. The incorporation of new computational procedures such as machine learning, neural networks or in the future artificial intelligence is very likely to facilitate and improve these predictive models. Moreover, care may be taken to combine the information provided by MST markers in multilayer data studies with other information from water uses in each geographic region (drinking water consumption, industrial use, agricultural irrigation and livestock, recharge of aquifers, reuse of wastewater, etc.) and hydrological and geological data among others that will allow to have a holistic view of water resources and their exploitability by different agents and uses.

On the other hand, MST studies in recent years are exploring whether the available MST markers can provide significant contributions to the control of waterborne pathogens, the transmission of antibiotic resistance through the water cycle, and to complement microbial quantitative risk assessments. It cannot be ruled out that some of the present MST markers, or others that may appear in the future, may play a key role in these topics.

New contributions from metagenomic and proteomic techniques may, in the not-too-distant future, be new tools for improving the specificity and sensitivity of MST markers or for discovering new ones that provide greater accuracy in operational applications of these MST markers.

## Author Contributions

MG, AB, and GR prepared the call for this Research Topic, edited the manuscripts that were submitted, and wrote this editorial. All authors contributed to the article and approved the submitted version.

## Conflict of Interest

The authors declare that the research was conducted in the absence of any commercial or financial relationships that could be construed as a potential conflict of interest.

## Publisher's Note

All claims expressed in this article are solely those of the authors and do not necessarily represent those of their affiliated organizations, or those of the publisher, the editors and the reviewers. Any product that may be evaluated in this article, or claim that may be made by its manufacturer, is not guaranteed or endorsed by the publisher.

